# A new brand for Disease Models & Mechanisms and The Company of Biologists

**DOI:** 10.1242/dmm.024067

**Published:** 2015-12-01

**Authors:** Rachel Hackett, O. Claire Moulton, Ross L. Cagan

**Affiliations:** 1 Disease Models & Mechanisms, The Company of Biologists, Bidder Building, Station Road, Histon, Cambridge CB24 9LF, UK; 2 Icahn School of Medicine at Mount Sinai, Annenberg 25-40, Campus Box 1020, 1468 Madison Avenue, New York, NY 10029, USA

## Abstract

**Summary:** The launch of a new brand and website for DMM.

Regular readers of Disease Models & Mechanisms (DMM) during 2015 will have noticed some changes to the journal. This is part of the gradual implementation of a new Company brand and migration to a new, better, web platform. This culminated in October with the launch of a new website for The Company of Biologists (www.biologists.com), and a brand new look and feel for the DMM website (dmm.biologists.org) and the articles it publishes (see Fig. 1). The new DMM website (and those of its sister journals Development, Journal of Cell Science, Journal of Experimental Biology and Biology Open) is the result of a mammoth project to ensure that users have an enhanced experience when visiting our pages. The site is easier to navigate and uncluttered, making it even quicker to search and find the content you need. We hope it looks good too.

**Fig. 1.**
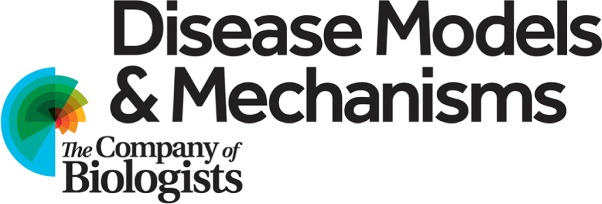
**The Company of Biologists and DMM: supporting biologists, inspiring biology.**

Although our five journals are well known, fewer people are aware of the other areas of support that The Company of Biologists brings to the biological community. Our new brand will help us to increase the awareness of our work, and strengthen the links between our journals and charitable activities.

## The Company of Biologists

The Company was created in 1925 by the eminent zoologist George Parker Bidder III to save an important but struggling journal – The British Journal of Experimental Biology. Further publications soon joined the fold, turning The Company of Biologists into a specialist publisher, but one with a mission to support biological research and discovery. In 1952, The Company of Biologists became a registered UK charity, using its profits to support scientific meetings, workshops, societies and grants.

## Charitable activities

As a charity, The Company of Biologists uses the surplus it generates for the benefit of biology and the biological community. The Company has made substantial contributions to the community, using funds to organise and facilitate scientific meetings, build and develop communities of biologists, assist the activities of specialist societies, and give financial support to young researchers. This investment is overseen by the Board of Directors, who give their time to the Company without payment. They are experienced senior scientists from a range of life science and clinical research backgrounds, who believe in the importance of what the Company does and who are dedicated to furthering its influence.

In 2010, the first in an ongoing series of Company-funded workshops took place (http://www.biologists.com/workshops/). These small workshops are carefully organised to provide leading experts and early career scientists from a diverse range of scientific backgrounds with an inspiring environment for the cross-fertilisation of interdisciplinary ideas. DMM Editor-in-Chief Ross Cagan is hosting a workshop on ‘Rethinking Cancer’ in November 2016. The aim of the workshop is to bring cancer experts together with experts in engineering, mathematics and computational biology, entrepreneurs and smart thinkers from the arts to help rethink a challenging and important health problem. We plan to present a series of commentaries and Editorials from the workshop in future issues of DMM. If you would love to host such a meeting, but don't have time to organise it or raise the funding, please contact us (http://www.biologists.com/workshops/propose-new-workshop/). The feedback we receive from attendees and organisers is overwhelmingly positive; you won't regret it!

DMM offers Travelling Fellowships to postgraduate students and postdoctoral fellows wishing to make collaborative visits to other research laboratories. Each application is reviewed by the Editor-in-Chief. We receive very positive feedback from recipients, who often find their careers taking exciting new directions following their visit (see Box 1 for one of our success stories). In October 2015, we launched the ‘DMM Travel Grant’ as something of an experiment in community funding. DMM Travel Grants are aimed at early career scientists wanting to attend meetings and courses relating to the areas of research covered by DMM, restricted during the trial to travel in 2015. Despite the relatively short time limit, we have had many applications and have very much enjoyed awarding these grants to some excellent applicants.

**Box 1. A DMM Travelling Fellowship story: Building lasting connections**

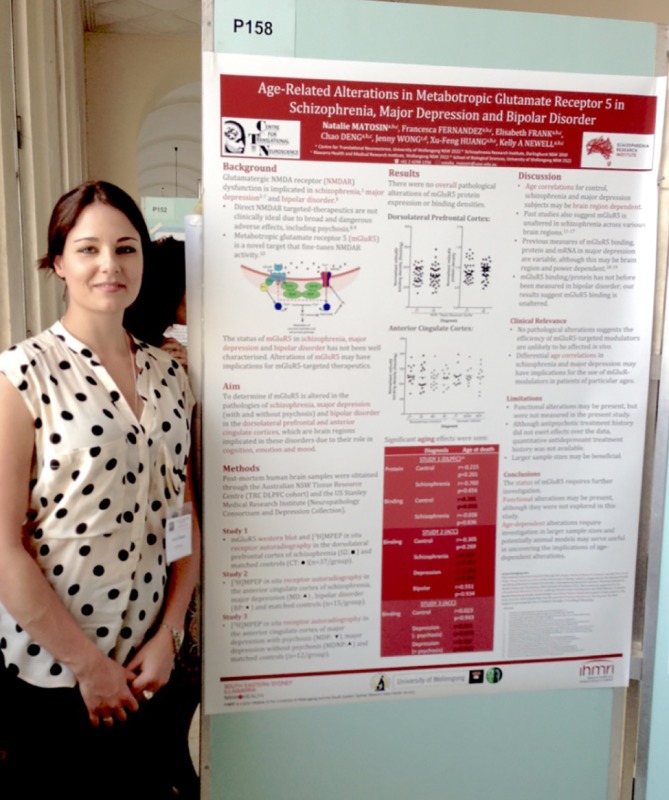

A Travelling Fellowship from DMM gave Natalie Matosin a unique opportunity to join a project within the Schmidt group at the Max Planck Institute of Psychiatry. The project built on the group's work on the role of mGluR5/Homer1 linkages in animal models of stress-related neuropsychiatric disorders.

This opportunity enabled Natalie not only to collaborate with the ‘Neurobiology of Stress' Schmidt group on their paper, but also to learn the techniques she required to complete projects at her home institution (University of Wollongong, Australia).

Together with Dr Klaus Wagner and Dr Nils Gassen, Natalie was able to complete a co-immunoprecipitation (Co-IP) of the mGluR5/Homer1 linkages in brain tissue from mice subjected to the chronic social defeat stress paradigm. Furthermore, with analysis of the literature and exploratory experimentation, she discovered a new signalling partner with which mGluR5 interacts.

The experience helped Natalie to learn many different experimental techniques, both within and beyond her immediate laboratory group. This included Co-IP, membrane fractionating, western blotting, immunohistochemistry and *in situ* hybridisation. Also, she was able to increase her understanding of animal handling and behavioural experimentation, and to join guest lectures to broaden her knowledge.

This opportunity exposed Natalie to new ideas and built the foundation of future collaboration with the Schmidt group at the Max Planck Institute of Psychiatry. It also helped Natalie to secure a post-doctoral position in Munich and the publication of her research.

## Reducing the pain to publish

In 2011, discussions among the Directors of The Company of Biologists became focused on the ‘pain to publish’ experienced by authors – and whether the Company should do something to help lessen that pain. We conducted a survey of our authors, editors and reviewers; the results *confirmed* the challenges faced by authors in finding a home for their papers. Later in 2011, a call for papers went out for the Company's newest journal Biology Open (BiO; bio.biologists.org) (Hunt and Moulton, 2012). BiO is an online Open Access journal that publishes peer-reviewed original research across all aspects of the biological sciences. Since its launch, BiO has published nearly 600 good-quality papers that have been accepted on the basis that they are technically sound and their conclusions are supported by the data shown – rather than on the perceived importance of the findings. What does this mean for DMM authors? Papers rejected from DMM can be transferred to BiO, strictly with the authors’ approval, using a one-click transfer mechanism. Under this service, any existing referees’ reports from the original peer review can also be passed to BiO, for a more-rapid publication decision. There is no need to reformat the article, which will be assessed using different criteria to DMM by research-active scientists. We hope that this enables authors to avoid additional rounds of submission and review (see Box 2 for recent BiO papers of interest to the DMM community). BiO is indexed by Scopus, Web of Science, PMC and PubMed, and has excellent Open Access credentials.

**Box 2. Recently published BiO papers of interest to the DMM community**

**Kazuhide S. Okuda, June Pauline Misa, Stefan H. Oehlers, Christopher J. Hall, Felix Ellett, Sultan Alasmari, Graham J. Lieschke, Kathryn E. Crosier, Philip S. Crosier and Jonathan W. Astin**. (2015). A zebrafish model of inflammatory lymphangiogenesis. *Biology Open***4**, 1270-1280.

**Alexandria Voigt, Lida Esfandiary and Cuong Q. Nguyen**. (2015). Sexual dimorphism in an animal model of Sjögren's syndrome: a potential role for Th17 cells. *Biology Open***4**, 1410-1419.

**Barbara Dhooghe, Charlotte Bouckaert, Arnaud Capron, Pierre Wallemacq, Teresinha Leal and Sabrina Noel**. (2015). Resveratrol increases F508del-CFTR dependent salivary secretion in cystic fibrosis mice. *Biology Open***4**, 929-936.

**Noriko Wakabayashi-Ito, Rami R. Ajjuri, Benjamin W. Henderson, Olugbenga M. Doherty, Xandra O. Breakefield, Janis M. O'Donnell and Naoto Ito**. (2015). Mutant human torsinA, responsible for early-onset dystonia, dominantly suppresses GTPCH expression, dopamine levels and locomotion in *Drosophila melanogaster*. *Biology Open***4**, 585-595.

**Colleen Valdez, Reese Scroggs, Rachel Chassen and Lawrence T. Reiter** (2015). Variation in Dube3a expression affects neurotransmission at the *Drosophila* neuromuscular junction. *Biology Open***4**, 776-782.

## Supporting biologists, inspiring biology

We hope that this Editorial has given you a clearer idea of the ethos and activities of The Company of Biologists. We exist to support biologists and inspire advances in biology. It is this aim that underpins the decisions that the Board of Directors make and the excellence we strive for in our publications. DMM's authors, reviewers and editors are all an essential component of this, and we hope that you will continue to support DMM and The Company of Biologists in the future.
